# The Effect of a Verbal Cognitive Task on Postural Sway Does Not Persist When the Task Is Over

**DOI:** 10.3390/s21248428

**Published:** 2021-12-17

**Authors:** Kayla Bohlke, Xiaonan Zhu, Patrick J. Sparto, Mark S. Redfern, Caterina Rosano, Ervin Sejdic, Andrea L. Rosso

**Affiliations:** 1 Department of Bioengineering, Swanson School of Engineering, University of Pittsburgh, Pittsburgh, PA 15260, USA; mredfern@pitt.edu; 2 Department of Epidemiology, School of Public Health, University of Pittsburgh, Pittsburgh, PA 15260, USA; xiz97@pitt.edu (X.Z.); rosanoc@edc.pitt.edu (C.R.); alr143@pitt.edu (A.L.R.); 3 Department of Physical Therapy, School of Health and Rehabilitation Sciences, University of Pittsburgh, Pittsburgh, PA 15260, USA; psparto@pitt.edu; 4 The Edward S. Rogers Department of Electrical and Computer Engineering, Faculty of Applied Science and Engineering, University of Toronto, Toronto, ON M5S 1A1, Canada; ervin.sejdic@utoronto.ca; 5 Research & Innovation Department, North York General Hospital, Toronto, ON M2K 1E1, Canada

**Keywords:** accelerometry, balance, dual-task, older adults, posture

## Abstract

Dual-task balance studies explore interference between balance and cognitive tasks. This study is a descriptive analysis of accelerometry balance metrics to determine if a verbal cognitive task influences postural control after the task ends. Fifty-two healthy older adults (75 ± 6 years old, 30 female) performed standing balance and cognitive dual-tasks. An accelerometer recorded movement from before, during, and after the task (reciting every other letter of the alphabet). Thirty-six balance metrics were calculated for each task condition. The effect of the cognitive task on postural control was determined by a generalized linear model. Twelve variables, including anterior–posterior centroid frequency, peak frequency and entropy rate, medial-later entropy rate and wavelet entropy, and bandwidth in all directions, exhibited significant differences between baseline and cognitive task periods, but not between baseline and post-task periods. These results indicate that the verbal cognitive task did alter balance, but did not bring about persistent effects after the task had ended. Traditional balance measurements, i.e., root mean square and normalized path length, notably lacked significance, highlighting the potential to use other accelerometer metrics for the early detection of balance problems. These novel insights into the temporal dynamics of dual-task balance support current dual-task paradigms to reduce fall risk in older adults.

## 1. Introduction

One third of people aged 65 and older fall each year, accounting for the majority of injury-related hospitalizations and deaths in older adults [1] and costing $500 billion annually in the US [2]. Falls are associated with decreased independence and lower life expectancy [1]. Older adults are more likely to fall when balance deficits are present. Postural control, i.e., the control of bodily position to maintain balance, was previously considered a relatively automatic process. However, dual-task studies have shown that postural control is altered during various cognitive tasks, indicating that postural control can require demonstrable attentional resources [3]. Additionally, the automaticity of postural control can decrease with age, leading to greater attentional demand to compensate. Cognitive tasks requiring more attention may cause competition for neural resources and lead to postural control disruptions [4]. It is well-known that cognitive function, particularly attention, also declines with age [5]. These age-related changes in cognition and postural control contribute to increased fall risk in older adults [3,6], but this relation is not well understood. Many dual-task studies have examined postural stability using a secondary task that requires some information processing. Changes in performance determine how much interference exists between the attentional requirements of the two tasks. The severity of these balance performance changes are highly variable and have shown conflicting results [3]. Additionally, the temporal dynamics of cognitive interference on postural control are currently unknown. Therefore, further research is necessary to clarify how and the extent to which cognitive tasks may affect postural control to reduce fall risk.

Dual-task studies of postural control of older adults have focused on effects during task performance [7,8,9] and the impact of various interventions [10,11]. To date, dual-task effects on postural control after task performance have not been examined. Commonly, dual-task paradigms will randomize the order of single-task and dual-task conditions [7,8,10,11]. If there are carry-over effects on postural control, single-task balance data collected after dual-task conditions could be biased and not represent true single-task measurements. Persistent effects also could have implications for fall risk, as an individual’s balance would be of concern not only while performing another task, but also for some time afterwards. The research presented in this paper is a secondary analysis of data collected for gait experiments. This study specifically investigated the time course of dual-task interference from pretask, during task, and immediate post-task on postural control performance by measuring center of mass accelerations. Previous studies have quantified balance using accelerometry, but the main outcomes are often limited—with root mean square, normalized path length, and sample entropy as some of the most common measurements [12]. Additional features in time, frequency, time-frequency, and information theory domains may provide important balance information that traditional measurements fail to capture. Thus, a comprehensive list of novel accelerometry features was extracted to describe standing balance with and without a dual-task. Compared to the current measurement techniques, such as force plates and motion capture systems, assessing balance using a single accelerometer could provide clinicians with a more accessible, affordable, and portable measurement tool. The goal of this study was twofold: (1) conduct an exploratory, descriptive analysis of balance performance accelerometry measures to find which measures are potentially useful for balance assessment using a single accelerometer; and (2) test the hypothesis that performing a verbal cognitive task alters postural control during the task and once completed. We hypothesized that postural stability would (A) change during the cognitive task and (B) fail to return to baseline levels after the cognitive task was completed. We tested our hypotheses by comparing accelerometry features from the pretask period (baseline) to those from (A) the cognitive task period and (B) the post-task period.

## 2. Materials and Methods

### 2.1. Subjects

This study includes data from 52 subjects from two different studies using the same experimental protocol. Twenty-eight older adults (M = 13, F = 15, 75 ±  6 years, range: 67–87 years) were recruited from a study of amyloid deposition in cognitively healthy older adults [13]. Primary inclusion criteria were at least 65 years old, no current or history of neurological or psychiatric disease, no history of stroke, magnetic resonance imaging (MRI) eligible, and able to walk unassisted. Twenty-four older adults (M = 9, F = 15, 74 ± 6 years, range: 68–91 years) were recruited from a longitudinal study of risk for mild cognitive impairment [14]. Primary inclusion criteria were at least 65 years old, no dementia, MRI and positron emission tomography eligible, and able to walk unassisted. This was a fixed sample derived from existing data that were developed for gait outcomes. Subjects between studies were compared on age, gait speed, and sex and the data were found to be similar so that the two data sets could be combined. The IRB of the University of Pittsburgh approved these procedures and all subjects gave informed consent.

### 2.2. Dual-Task Procedures

This research is a secondary analysis of data that were collected for a gait study. All subjects performed a mobility protocol described in detail in Hoppes et al. 2020 [15]. This study focused on standing portions of the protocol which were performed consecutively: (1) quiet standing (pretask), (2) standing and cognitive task (task), and (3) quiet standing (post-task). Each task was 20 s long. The cognitive task was reciting every other letter of the alphabet starting with the letter ‘B’. Subjects were instructed to start back at ‘B’ if they complete the alphabet before the 20 s is over. This task was selected to parallel carrying a conversation [15]. Subjects performed one set of the consecutive tasks twice during each of four walking trials for a total of eight completed sets. For each walking trial, subjects completed two loops of a track, with the standing sets randomly interspersed among walking on even and uneven surfaces as single- or dual-task conditions with the same cognitive task. Subjects were instructed to stand quietly; no instructions were given regarding foot placement during standing or task prioritization.

Alphabet performance, a measure of cognitive ability, was quantified by dividing the number of correct letters by the duration of the cognitive task (20 s) and was averaged over the eight trials. To quantify a participant’s general physical function, gait speed was measured by timing subjects on a flat 15-m straight pathway. Four trials were measured in m/s and then averaged per subject.

### 2.3. Postural Control Metrics

A tri-axial accelerometer (ActiGraph wGT3X, ActiGraph LLC, Pensacola, FL, USA) placed over the L3 segment of the lumbar spine measured linear accelerations of the approximated center of mass (CoM) [16] in the medial-lateral (ML), vertical (V), and anterior–posterior (AP) axes. We chose to include vertical signals since these were exploratory analyses and the vertical direction is not typically represented in literature on balance. Accelerometry has been validated to evaluate postural control performance [17]. For the pretask and post-task conditions, the signals were trimmed to avoid overlap with walking tasks. The last 15 s of the pretask (PRE) condition signal and the first 15 s of the post-task (POST) condition signal were used. All 20 s of the task (COG) condition signal were used.

**Figure 1:**
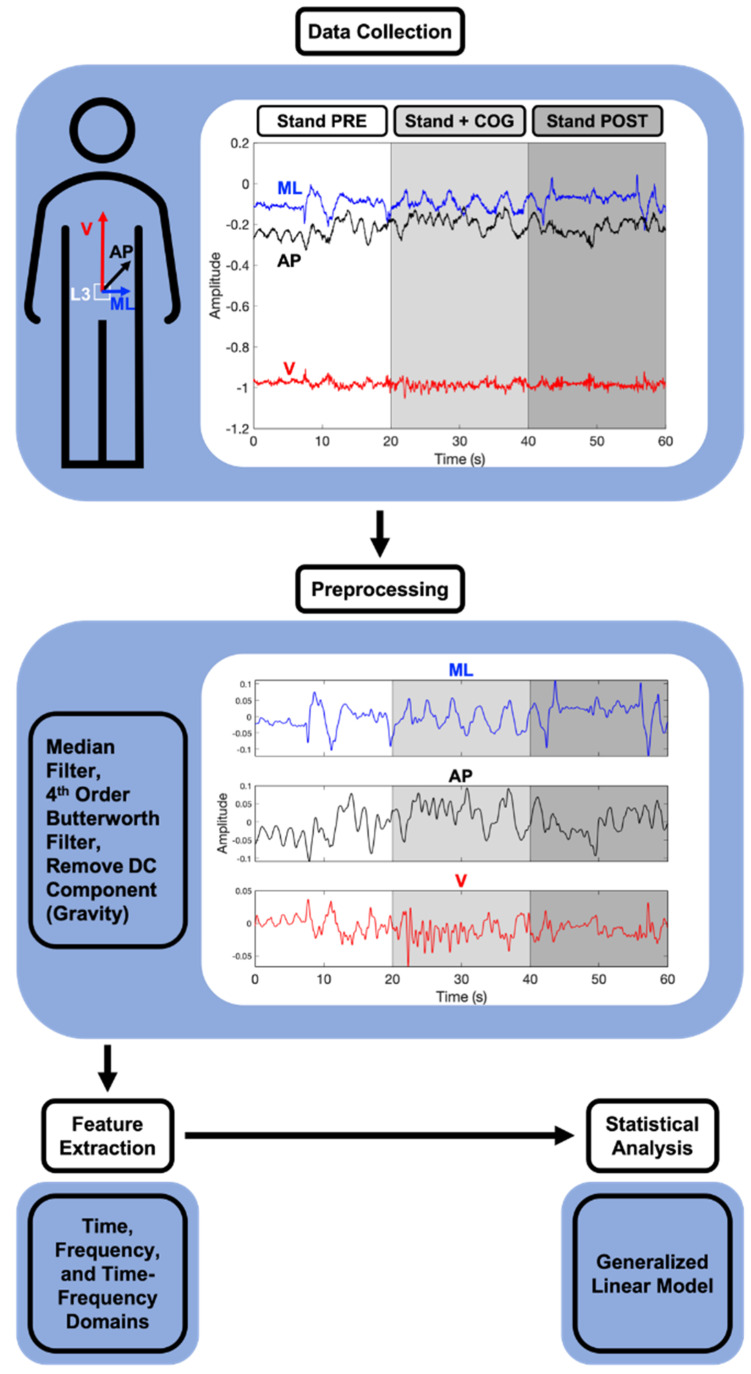
Flow diagram of the data processing pipeline to extract accelerometry features.

Acceleration signals were sampled at 100 Hz for 39 subjects; the remaining 14 subjects were sampled at 30 Hz due to technical issues. For those measured at 30 Hz, the signals were up-sampled to 100 Hz by first zero-padding the signals and then using a finite-impulse response anti-aliasing low pass filter method that employs a Kaiser window. This method preserves the frequency content of the signals [18,19]. Impulse-like artifacts were then removed using a median-filter [20]. The signals were then processed with a 4th order, low-pass Butterworth filter with a cutoff frequency of 2 Hz [21]. The effect of gravity was removed using coordinate transformations to account for accelerometer tilt [22] and subtracting the mean from each signal [20]. Root-mean-square (RMS) and normalized path length (NPL) were selected as primary postural control features [17,23,24]. In addition to the two time-domain measures, three frequency, one time-frequency, three statistical, and three information theory features were extracted based on their use in gait accelerometry analysis [25]. Altogether, 12 different signal processing features were implemented. These features were extracted from all three directional signals for each task and averaged over the eight trials for a total of 36 signal features per subject. All signal processing was done using custom Matlab code (version 2020a, MathWorks, Natick, MA, USA). The data processing pipeline is outlined in Figure 1. Definitions, descriptions, and acronyms of the different features are in Table A1.

Any signal with more than 2 s of signal drop (consecutive 0 values) was deemed to be of insufficient signal quality and removed from the analysis. Of the 64 subjects, 12 did not have sufficient signal quality for each of the three dual-task conditions (PRE, COG, POST) and were removed from analysis. Subjects with poor signal quality were compared to included subjects to identify any systematic differences between groups. Age, gait speed, alphabet performance, and sex were all examined in relation to amount of signal drop using scatter plots and none showed trends of difference between those included and excluded.

### 2.4. Statistical Analysis

The effect of cognitive task on postural control was determined by a generalized linear regression model with the random effect of person to account for repeated measures. Model fit was tested by assessing residual normality. F-values were reported for global differences among PRE, COG, and POST conditions. β-estimates from the model showed differences between pairs of conditions. Significance was determined at α=0.05 . Accelerometry features with significant global differences among conditions were then examined for associations with age, gait speed, and alphabet performance using Pearson correlations. Specifically, values obtained during the PRE condition, change from PRE to COG, and change from PRE to POST were assessed. To account for multiple comparisons, the Dunn-Sidak correction for significance was calculated using an initial value of α=0.05 , resulting in a corrected value of α=0.0004 . The general linear regression model was implemented using SAS software (version 9.4, SAS Institute, Gary, NC, USA) and the correlations were computed using Matlab (version 2020a, MathWorks, Natick, MA, USA).

## 3. Results

Table 1 summarizes descriptive characteristics of the 52 included subjects. Average age for the combined sample was 75 ± 6 years (range of 67–91). Average gait speed was 1.03 ±  0.22 m/s and average alphabet performance was 0.61 ± 0.18 correct letters/s.

The 36 accelerometry variables are summarized in Table 2. For our primary postural control features, no significant differences among conditions were found for RMS or NPL in any direction. Additionally, synchronization index (SI) and skewness (SKEW) lacked significant differences.

The following variables did demonstrate statistically different values among the three conditions (*p* < 0.05): centroid frequency (CFR), peak frequency (PFR), entropy rate (ENTR), wavelet entropy (WE), and kurtosis (KURT) in the AP direction; PFR, ENTR, and WE in the ML direction; Lempel-Ziv complexity (LZ) in the V direction; bandwidth (BND) in all directions; and cross-correlations (CORR) between the ML and AP signals and between the AP and V signals. PFR in the ML direction showed differences between the PRE and POST task. WE in the AP direction showed that the three conditions were not equal, but no significant differences were found between PRE and COG or PRE and POST. The remaining 12 variables have differences between the PRE and COG task (Table 3). Specific F-values and *p*-values can be found in Supplementary Materials (Table S1). Box plots for the significant variables can be found in Supplementary Materials (Figure S1). Of the 14 significant variables, only AP BND during the PRE condition showed a significant, moderate correlation with average gait speed (r = 0.490, *p* = 0.0002) after using the Dunn-Sidak correction for multiple comparisons ( α=0.0004 ).

## 4. Discussion

We found that 14 out of 36 accelerometry features differed during a standing dual-task protocol. Differences were observed from pretask to during task and returned to pretask levels during the post-task phase for all variables except for PFR in the ML direction and WE in the AP direction.

RMS and NPL are some of the most common signal features extracted from force plate measurements, and previous research has shown that accelerometry measures correlate well with those from force plates [17]. RMS is a measure of variability of signal amplitudes relating to the dispersion of the amount of sway. NPL is a measure of how fast the person is moving, giving an indication of how fast and how often the person is correcting their balance. No significant differences were found among conditions for RMS or NPL in any direction. The lack of significant differences among conditions could be a reflection of the time variance of postural sway [26]. Even though the traditional features are not informative for the balance protocols used in this study, we were able to detect differences in several other features.

CFR and PFR in the AP direction and PFR in the ML direction showed significant differences among the conditions. CFR is the frequency at which the power in the spectrum above that frequency is equal to the power in the spectrum below that frequency. PFR is the frequency at which the highest amount of power is attributed. Average CFR and PFR in the AP direction decreased during COG condition but generally returned to baseline during POST condition. This may reflect that subjects while under cognitive load are exhibiting slower oscillations. The shift to lower frequencies may also indicate a less stiff sway [27], as attention is shifted to the cognitive task during the COG condition. Conversely, increases in ML PFR in the POST condition compared to the PRE condition may indicate and increased postural stiffening. BND for each direction was lower during the COG task than the PRE task and returned to baseline by POST task. Smaller BND values indicate a narrower frequency response to maintain balance during the COG task. These BND results could be interpreted as subjects being less adaptable during the COG task as they are limiting their potential balance responses.

ENTR measures the regularity of the signals by examining the relatedness of consecutive points, with higher values being associated with higher regularity and lower values with randomness [25]. ENTR in the ML and AP directions showed increases—i.e., higher regularity—during COG condition and returned to baseline during POST condition. Higher regularity is associated with less automatic control and more ineffective postural strategies [28] as attentional resources are diverted to the cognitive task [29]. Younger adults usually show decreased regularity during cognitive tasks, as the task pulls attention from balance and increases automaticity and efficiency [30]. Thus, our could results indicate that the attentional resources diverted away from postural control may be necessary to compensate for the postural control automaticity that is lost with age. WE in the ML and AP directions also showed significant differences among conditions. WE measures how disordered a signal is by measuring the contribution of different frequency bands on the wavelet representation of the signal. Higher WE values indicate more disordered, random signals [25]. Results from the linear model showed that WE AP in the three conditions were not equal, but no significant differences were found between PRE and COG conditions nor between PRE and POST conditions. Due to the limitations of the model, we did not test for differences between COG and POST conditions. In contrast, WE ML had significant increases during the COG condition, indicating higher disorder and randomness, before returning to baseline. It is important to note that ENTR and WE analyze randomness on different scales. ENTR measures randomness between consecutive points which is a more local metric; while WE is a more global metric and measures randomness across time-frequency bands.

CORR measures the similarity between two signals. CORR ML-AP and CORR AP-V showed decreased values during the COG condition and similar values for POST and PRE conditions. Decreased CORR means the signals were less coupled during the COG condition but they returned to baseline during the POST condition. KURT is a statistical metric that quantifies how spread out signal amplitudes are from the mean. KURT in the AP direction was significantly higher during the COG condition compared to the PRE condition. Higher values mean more peaked distributions (fewer outliers) and indicate less variable sway. LZ measures the predictability of the signal, and higher values indicate less predictable, more complicated signals [25]. LZ in the V direction was significantly higher during COG condition with a return to baseline during the POST condition, pointing to more complex postural control while under cognitive load.

The lack significant findings in our primary outcomes (RMS and NPL) and the slight disagreement between the significant features in terms of returning to baseline levels of balance performance could indicate that the dual-task conditions were not difficult enough to elicit strong differences in this relatively healthy sample [3]. Additionally, the changes in postural control performance may be too minute to strongly alter balance performance. On the other hand, the RMS and NPL results may show a floor effect meaning that the other accelerometry metrics may be more sensitive to changes in balance.

We are unable to determine whether the changes we observe represent maladaptive effects on balance control (i.e., cognitive interference) or other adaptive strategies. For example, higher complexity and randomness in the signal may reflect better online adjustments, allowing the individual to adapt to perturbations more easily. LZ V points to higher complexity, ENTR ML and AP point to higher local regularity, and WE ML points to higher global randomness. Different explanations for changes in postural control performance in older adults, cognitive task difficulty, stiffening method and signal-to-noise ratio, may support our varied results.

Several neuromotor mechanisms potentially underly the observed results. Cognitive mechanisms suggest that a concurrent information processing task requires cognitive resources normally used to control posture, particularly executive functions [31,32,33]. Another potential mechanism is generalized slowing with aging that can account for changes in processes requiring cognition [31,34]. Dual-task postural responses can show increases in sway and reduced sway, depending upon the cognitive and postural task. A number of studies have shown increased sway amplitude with a concurrent cognitive task [3,4]. However, older adults can show reduced postural sway under dual-task [35]. A potential biomechanical mechanism is postural stiffening, characterized by reduced sway distance and higher frequency components, which indicates more frequent adjustments [36]. Under these conditions, the cognitive task performance does not suffer and postural control processing is believed to become automatic, thus less interference of shared associated brain regions occurs [37]. Alternatively, deficits in postural control in older adults may be due to decreased signal-to-noise ratio from declines in sensory systems and muscular strength. This decreased signal-to-noise ratio would then require recruitment of more neural resources to make up for reduced sensitivity of the sensory inputs and reduced functionality of the motor outputs [37].

The trend of some variables indicating balance improvement and others indicating deficits may be supported by several of these theories. Some subjects may have been stiffening and improving their postural control performance [38]; others may have had deficits in sensory integration [31] that resulted in poorer performance when attention was deviated. Some subjects may have been more likely to use hip strategy than ankle strategy and vice versa [39,40].

For this study, the cognitive task did not show carry-over effects. The changes that occurred in postural sway during the concurrent cognitive task returned to their pretask levels once the cognitive task had been completed. Previous studies have not examined the time course of cognitive effects on postural control. The ramifications for these results are twofold. Firstly, our results indicate that changes in postural stability are due specifically to the cognitive task (i.e., once removed, the changes in sway return to baseline). This is important not only for exploratory dual-task studies but also for interventions that rely on single- and dual-task performance measurements to evaluate the effectiveness of the intervention. Secondly, this verbal cognitive task was intended to mimic attentional demands of everyday activities such as carrying on a conversation, so these results have important implications for fall risk. While performing a cognitive task may alter balance in a way that could lead to higher incidence of falls, our results indicate that subjects do not have continued diminished capabilities after the task. Confirming that effects from cognitive tasks on postural stability do not persist may provide some relief for individuals with fear of falling, a factor that contributes to increased fall risk. Additionally, this information could guide caregivers to limit multitasking in patients with high fall risk.

The comprehensive list of accelerometry features in this study includes many that are not common in the literature for balance assessments (e.g., bandwidth, wavelet entropy). Our results show that these accelerometer metrics identify changes that do not show up on traditional force plate measures. In the interest of early detection, accelerometry may be a more sensitive way to look for very early balance problems. Early detection of balance problems could serve as a biomarker for neurodegeneration because balance deficits seem to predate neurodegeneration [6]. Accordingly, detecting balance changes early enough is imperative to develop effective interventions for preventative care or treatment. Integrating accelerometers into balance assessments would provide clinicians with objective and sensitive measurements. Accelerometers also provide an opportunity to expand accessibility of balance assessments due to their portability and commercial availability. Not only would more clinics be capable of obtaining this technology, but balance assessments could be administered in community settings for those who are unable to travel to receive healthcare services.

Some limitations were due to experimental setup being optimized for gait and not for standing postural control. The length of each trial was only 20 s. Healthy older adults have more varied postural control during the first 30 s of quiet standing before leveling out [41]. Additionally, there were problems with signal drop at low levels of activity due to an “idle sleep mode” that caused the accelerometers to enter low battery mode. Signals that contained more than 2 s of dropped signal were removed from analysis. This data removal could have skewed the data towards more variant balance performance. Frequency domain variables provided limited information due to low frequency resolution and length of tasks. With signal lengths of 1200 to 1700 points, identifying specific frequencies is more challenging. This study did not account for potential effects of vocalization on postural sway. Vocalization affects mean sway frequency but not mean sway velocity or sway area [42]. Thus, our frequency measures could have been altered by vocalization. In future studies, more challenging postural tasks, like single-leg standing or translational perturbations, and more attentionally demanding, nonverbal cognitive tasks could be used to further explore potential for carry-over effects on posture.

Our study had several strengths. This is the first study to examine balance before, during, and after a cognitive task to evaluate the temporal dynamics of changes in postural control. Additionally, while most balance studies look at only a few outcomes, we extracted signal features from a variety of domains to provide a more comprehensive understanding of balance control. These novel insights in temporal dynamics and broader quantification of postural control will inform future dual-task experiments, diagnostic tests, and interventions that aim to improve balance.

## 5. Conclusions

Sustained alterations to postural control after completing recitation of alternating letters of the alphabet did not occur in healthy, older adults. These findings have important implications for dual-task paradigm design and for fall risk in older adults. With no threat to balance after the cognitive task, the focus of dual-task interference lies solely on the cognitive task condition. The lack of persistent effects on postural control after the secondary task indicates that an individual’s balance would only be of concern while performing another task.

## Figures and Tables

**Table 1:** Summary of demographic information and descriptive characteristics for subjects by study and combined.

Variable	Study 1 ^1^ (*n* = 28)	Study 2 ^2^ (*n* = 25)	Total (*n* = 53)
Female (*n*, %)	15, 54%	15, 63%	30, 58%
White (*n*, %)	21, 75%	23, 96%	44, 85%
Age (years)	75 ± 6	74 ± 6	75 ± 6
Gait Speed (m/s)	0.98 ± 0.13	1.10 ± 0.28	1.03 ± 0.22
Alphabet Performance (correct letters/s)	0.63 ± 0.22	0.58 ± 0.11	0.61 ± 0.18

^1^ Study of amyloid deposition in cognitively healthy older adults; ^2^ Longitudinal study of risk for mild cognitive impairment.

**Table 2:** Averaged raw values across task type for each feature in each direction. * (medium gray) Differences are significant between PRE and COG conditions; † (dark gray) Differences are significant between PRE and POST conditions; ‡ (light gray) PRE, COG, and POST are not all equal.

		PRE	COG	POST
Feature	Direction	Mean ± STD	Mean ± STD	Mean ± STD
**RMS** **(G)**	**ML**	0.011 ± 0.007	0.011 ± 0.005	0.009 ± 0.006
	**V**	0.003 ± 0.003	0.004 ± 0.003	0.003 ± 0.003
	**AP**	0.029 ± 0.021	0.028 ± 0.011	0.027 ± 0.015
**NPL** **(G/s)**	**ML**	0.023 ± 0.018	0.023 ± 0.011	0.019 ± 0.015
	**V**	0.011 ± 0.075	0.018 ± 0.021	0.013 ± 0.023
	**AP**	0.031 ± 0.017	0.038 ± 0.018	0.031 ± 0.019
**CFR** **(Hz)**	**ML**	0.47 ± 0.15	0.45 ± 0.17	0.52 ± 0.25
	**V**	1.10 ± 0.31	1.06 ± 0.28	1.13 ± 0.34
	AP *	0.29 ± 0.08	0.25 ± 0.07	0.29 ± 0.09
**PFR** **(Hz)**	**ML ^†^**	0.19 ± 0.11	0.17 ± 0.13	0.26 ± 0.26
	**V**	0.64 ± 0.41	0.81 ± 0.50	0.62 ± 0.47
	AP *	0.14 ± 0.06	0.08 ± 0.05	0.14 ± 0.09
**BND** **(Hz)**	ML *	0.92 ± 0.32	0.74 ± 0.26	0.95 ± 0.43
	V *	1.63 ± 0.73	1.00 ± 0.44	1.70 ± 0.66
	AP *	0.82 ± 0.27	0.69 ± 0.27	0.87 ± 0.34
**ENTR**	ML *	0.88 ± 0.015	0.90 ± 0.020	0.88 ± 0.009
	**V**	0.86 ± 0.030	0.86 ± 0.031	0.86 ± 0.030
	AP *	0.89 ± 0.009	0.91 ± 0.008	0.89 ± 0.010
**WE**	ML *	0.40 ± 0.23	0.57 ± 0.38	0.44 ± 0.26
	**V**	0.67 ± 0.32	0.77 ± 0.33	0.66 ± 0.38
	**AP ^‡^**	0.30 ± 0.18	0.37 ± 0.26	0.26 ± 0.17
**SI**	**ML-V**	0.86 ± 0.06	0.88 ± 0.06	0.86 ± 0.07
	**ML-AP**	0.87 ± 0.05	0.85 ± 0.05	0.87 ± 0.05
	**AP-V**	0.87 ± 0.06	0.88 ± 0.06	0.87 ± 0.07
**CORR**	**ML-V**	0.35 ± 0.08	0.32 ± 0.13	0.36 ± 0.09
	ML-AP *	0.42 ± 0.07	0.39 ± 0.09	0.45 ± 0.11
	AP-V *	0.37 ± 0.15	0.31 ± 0.11	0.37 ± 0.15
**SKEW**	**ML**	0.11 ± 0.69	−0.04 ± 1.07	−0.02 ± 0.97
	**V**	−0.63 ± 0.85	−0.63 ± 1.20	−0.60 ± 0.91
	**AP**	−0.06 ± 0.51	−0.04 ± 0.73	0.01 ± 0.50
**KURT**	**ML**	5.37 ± 2.74	7.36 ± 5.96	6.40 ± 6.20
	**V**	10.13 ± 6.30	9.57 ± 9.21	10.10 ± 6.60
	AP *	3.33 ± 0.90	3.89 ± 1.31	3.28 ± 1.16
**LZ**	**ML**	0.32 ± 0.04	0.31 ± 0.05	0.32 ± 0.04
	V *	0.32 ± 0.06	0.35 ± 0.05	0.31 ± 0.06
	**AP**	0.31 ± 0.04	0.30 ± 0.04	0.30 ± 0.05

**Table 3:** Summary of significant features from the generalized linear regression model. - (white) Indicates no significant differences. **△** (light gray) Indicates that the three conditions were not all equal. **✓** (medium gray) Indicates that the PRE condition was different from the COG condition. **✕** (dark gray) Indicates that the PRE condition was different from the POST condition.

Feature	ML	V	AP
**RMS**	-	-	-
**NPL**	-	-	-
**CFR**	-	-	**✓**
**PFR**	**✕**	-	**✓**
**BND**	**✓**	**✓**	**✓**
**ENTR**	**✓**	-	**✓**
**WE**	**✓**	-	**△**
**SI**	**(ML-V)** **-**	**(AP-V)** **-**	**(ML-AP)** **-**
**CORR**	**(ML-V)** **-**	**(AP-V)** **✓**	**(ML-AP)** **✓**
**SKEW**	-	-	-
**KURT**	-	-	**✓**
**LZ**	-	**✓**	-

## Data Availability

Data are available at request.

## Supplementary Materials

The following are available online at https://www.mdpi.com/1424-8220/21/24/8428/s1. Table S1: Summary of the results from the generalized linear regression model: F-value (*p*-value). Features in gray showed significant differences among the three conditions. * (medium gray) Differences are significant between PRE and COG conditions; † (dark gray) Differences are significant between PRE and POST conditions; ‡ (light gray) PRE, COG, and POST are not all equal. Figure S1: Box plots showing change from baseline for all significant variables. For each subject, average PRE values are subtracted from their averaged COG and POST values. Baseline, or initial values measured during the PRE condition, is indicated by the dashed line at 0. All variables except PFR ML and WE AP deviate during the COG condition and then return to baseline during the POST condition according to the results of the generalized linear regression model.

## Appendix A

**Table A1:** Acronym definitions and descriptions.

Acronym	Definition	Measurement	Connection to Balance
COG	Cognitive task	-	-
PRE	Quiet standing before cognitive task	-	-
POST	Quiet standing after cognitive task	-	-
ML	Medial-lateral signal	Linear acceleration left/right	-
V	Vertical signal	Linear acceleration up/down	-
AP	Anterior–posterior signal	Linear acceleration forward/backward	-
**Accelerometry features**			
RMS	Root mean square	Measure of spread (G)	Higher values indicate more sway
NPL	Normalized path length	Measure of speed (G/s)	Higher values indicate more distance traveled, thus more frequent adjustments and poorer postural control
CFR	Centroid frequency	Frequency that halves the power spectrum (Hz)	Lower values indicate poor postural control
PFR	Peak frequency	Frequency with the most power (Hz)	High values indicate more frequent postural adjustments and thus poorer postural control
BND	Bandwidth	Range of frequencies in the signal (Hz)	The larger the range, the more frequencies used to maintain balance
ENTR	Entropy rate	Measure of the regularity of the signal, index from 0 to 1	Values closer to 1 indicate high signal regularity; values closer to 0 indicate high signal randomness
WE	Wavelet entropy	Measure of signal disorder, randomness	Values closer to 0 indicate ordered signals; high values indicate disordered signals with equivalent contributions from most frequencies
SI	Cross entropy rate/Index of synchronization	Measure of signal predictability using past and present points from another signal, index from 0 to 1	Values closer to 1 indicate signals are highly synchronized
CORR	Cross correlation	Measure of similarity between two signals, index from 0 to 1	Values closer to 1 indicate higher agreement between signals
SKEW	Skewness of signal	Measure of asymmetry of amplitudes about the mean	Higher absolute values (positive or negative) indicate more asymmetry in postural control
KURT	Kurtosis of signal	Measure of how spread out the amplitudes are from the mean	Higher values indicate more peaked distributions and thus less variable sway and fewer extreme outliers
LZ	Lempel-Ziv complexity	Measure of the complexity of the signal	Higher values indicate less predictable, more complex signals and better postural control
