# Correction: Bohlke et al. The Effect of a Verbal Cognitive Task on Postural Sway Does Not Persist When the Task Is Over. *Sensors* 2021, *21*, 8428

**DOI:** 10.3390/s23167218

**Published:** 2023-08-17

**Authors:** Kayla Bohlke, Xiaonan Zhu, Patrick J. Sparto, Mark S. Redfern, Caterina Rosano, Ervin Sejdic, Andrea L. Rosso

**Affiliations:** 1 Department of Bioengineering, Swanson School of Engineering, University of Pittsburgh, Pittsburgh, PA 15260, USA; mredfern@pitt.edu; 2 Department of Epidemiology, School of Public Health, University of Pittsburgh, Pittsburgh, PA 15260, USA; xiz97@pitt.edu (X.Z.); rosanoc@edc.pitt.edu (C.R.); alr143@pitt.edu (A.L.R.); 3 Department of Physical Therapy, School of Health and Rehabilitation Sciences, University of Pittsburgh, Pittsburgh, PA 15260, USA; psparto@pitt.edu; 4 The Edward S. Rogers Department of Electrical and Computer Engineering, Faculty of Applied Science and Engineering, University of Toronto, Toronto, ON M5S 1A1, Canada; ervin.sejdic@utoronto.ca; 5 Research & Innovation Department, North York General Hospital, Toronto, ON M2K 1E1, Canada

## 1. Text Correction

There was an error in the original publication [1]. In the Discussion, the interpretation of one of the variables was described incorrectly. The LZ variable was explained to show more predictable, less complicated signals with higher values, when the opposite is correct.

A correction has been made to Discussion, Paragraph 5:

CORR measures the similarity between two signals. CORR ML-AP and CORR AP-V showed decreased values during the COG condition and similar values for POST and PRE conditions. Decreased CORR means the signals were less coupled during the COG condition but they returned to baseline during the POST condition. KURT is a statistical metric that quantifies how spread out signal amplitudes are from the mean. KURT in the AP direction was significantly higher during the COG condition compared to the PRE condition. Higher values mean more peaked distributions (fewer outliers) and indicate less variable sway. LZ measures the predictability of the signal, and higher values indicate less predictable, more complicated signals [25]. LZ in the V direction was significantly higher during COG condition with a return to baseline during the POST condition, pointing to more complex postural control while under cognitive load.

A correction has been made to Discussion, Paragraph 7:

We are unable to determine whether the changes we observe represent maladaptive effects on balance control (i.e., cognitive interference) or other adaptive strategies. For example, higher complexity and randomness in the signal may reflect better online adjustments, allowing the individual to adapt to perturbations more easily. LZ V points to higher complexity, ENTR ML and AP point to higher local regularity, and WE ML points to higher global randomness. Different explanations for changes in postural control performance in older adults, cognitive task difficulty, stiffening method and signal-to-noise ratio, may support our varied results.

## 2. Error in Table

In the original publication [1], there was a mistake in Table A1 as published. The last variable in the table was incorrectly described as showing more predictable, less complicated signals when values are high. The directionality is the opposite, with high values indicating less predictable, more complex signals. The corrected Table A1 appears below.

The authors apologize for any inconvenience caused and state that the scientific conclusions are unaffected. This correction was approved by the Academic Editor. The original article has been updated.

## Figures and Tables

**Table A1:** Acronym definitions and descriptions.

Acronym	Definition	Measurement	Connection to Balance
COG	Cognitive task	-	-
PRE	Quiet standing before cognitive task	-	-
POST	Quiet standing after cognitive task	-	-
ML	Medial-lateral signal	Linear acceleration left/right	-
V	Vertical signal	Linear acceleration up/down	-
AP	Anterior–posterior signal	Linear acceleration forward/backward	-
**Accelerometry features**			
RMS	Root mean square	Measure of spread (G)	Higher values indicate more sway
NPL	Normalized path length	Measure of speed (G/s)	Higher values indicate more distance traveled, thus more frequent adjustments and poorer postural control
CFR	Centroid frequency	Frequency that halves the power spectrum (Hz)	Lower values indicate poor postural control
PFR	Peak frequency	Frequency with the most power (Hz)	High values indicate more frequent postural adjustments and thus poorer postural control
BND	Bandwidth	Range of frequencies in the signal (Hz)	The larger the range, the more frequencies used to maintain balance
ENTR	Entropy rate	Measure of the regularity of the signal, index from 0 to 1	Values closer to 1 indicate high signal regularity; values closer to 0 indicate high signal randomness
WE	Wavelet entropy	Measure of signal disorder, randomness	Values closer to 0 indicate ordered signals; high values indicate disordered signals with equivalent contributions from most frequencies
SI	Cross entropy rate/Index of synchronization	Measure of signal predictability using past and present points from another signal, index from 0 to 1	Values closer to 1 indicate signals are highly synchronized
CORR	Cross correlation	Measure of similarity between two signals, index from 0 to 1	Values closer to 1 indicate higher agreement between signals
SKEW	Skewness of signal	Measure of asymmetry of amplitudes about the mean	Higher absolute values (positive or negative) indicate more asymmetry in postural control
KURT	Kurtosis of signal	Measure of how spread out the amplitudes are from the mean	Higher values indicate more peaked distributions and thus less variable sway and fewer extreme outliers
LZ	Lempel-Ziv complexity	Measure of the complexity of the signal	Higher values indicate less predictable, more complex signals and better postural control
