# Supplementary material for: The Effect of a Verbal Cognitive Task on Postural Sway Does Not Persist When the Task Is Over
Source: Sensors (Basel). 2021 Dec 17;21(24):8428. doi: 10.3390/s21248428 (PMC8704561; doi:10.3390/s21248428)
Supplement: Supplementary file 1 [file sensors-21-08428-s001.zip › sensors-1474239-supplementary.pdf]

sensors

MDPI

Article

The Effect of a Verbal Cognitive Task on Postural Sway Does Not Persist When the Task Is Over

Kayla Bohlke [1,*], Xiaonan Zhu [2], Patrick J. Sparto [3], Mark S. Redfern [1], Caterina Rosano [2], Ervin Sejdic [4,5] and Andrea L. Rosso [2]

[1] Department of Bioengineering, Swanson School of Engineering, University of Pittsburgh, Pittsburgh, PA 15260, USA; mredfern@pitt.edu
[2] Department of Epidemiology, School of Public Health, University of Pittsburgh, Pittsburgh, PA 15260, USA; xiz97@pitt.edu (X.Z.); rosanoc@edc.pitt.edu (C.R.); alr143@pitt.edu (A.L.R.)
[3] Department of Physical Therapy, School of Health and Rehabilitation Sciences, University of Pittsburgh, Pittsburgh, PA 15260, USA; psparto@pitt.edu
[4] The Edward S. Rogers Department of Electrical and Computer Engineering, Faculty of applied Science and Engineering, University of Toronto, Toronto, ON M5S 1A1, Canada; ervin.sejdic@utoronto.ca
[5] Research & Innovation Department, North York General Hospital, Toronto, ON M2K 1E1, Canada
* Correspondence: ksb57@pitt.edu

Supplementary Materials:

Table S1. Summary of the results from the generalized linear regression model: F-value(p-value). Features in gray showed significant differences among the three conditions. * (medium gray) Differences are significant between PRE and COG conditions; † (dark gray) Differences are significant between PRE and POST conditions; ‡ (light gray) PRE, COG, and POST are not all equal.

Feature	ML	V	AP
RMS	1.74(.179)	1.27(.282)	0.25(.779)
NPL	1.20(.304)	2.28(.106)	2.20(.115)
CFR	1.88(.157)	0.86(.426)	5.61(.005)*
PFR	3.72(.026)†	2.65(0.75)	11.0(<.0001)*
BND	5.08(.007)*	20.18(<.0001)*	5.48(.005)*
ENTR	35.33(<.0001)*	0.17(.848)	133(<.0001)*
WE	4.55(.012)*	1.54(.217)	3.56(.031)‡
SI	ML-V 0.98(.376)	AP-V 0.31(.731)	ML-AP 2.19(.115)
CORR	ML-V 2.34(.100)	AP-V 3.27(.041)*	ML-AP 5.73(.004)*
SKEW	0.42(.657)	0.02(.978)	0.25(.781)
KURT	1.88(.156)	0.09(.911)	4.25(.016)*
LZ	1.15(.319)	6.02(.003)*	0.81(.448)

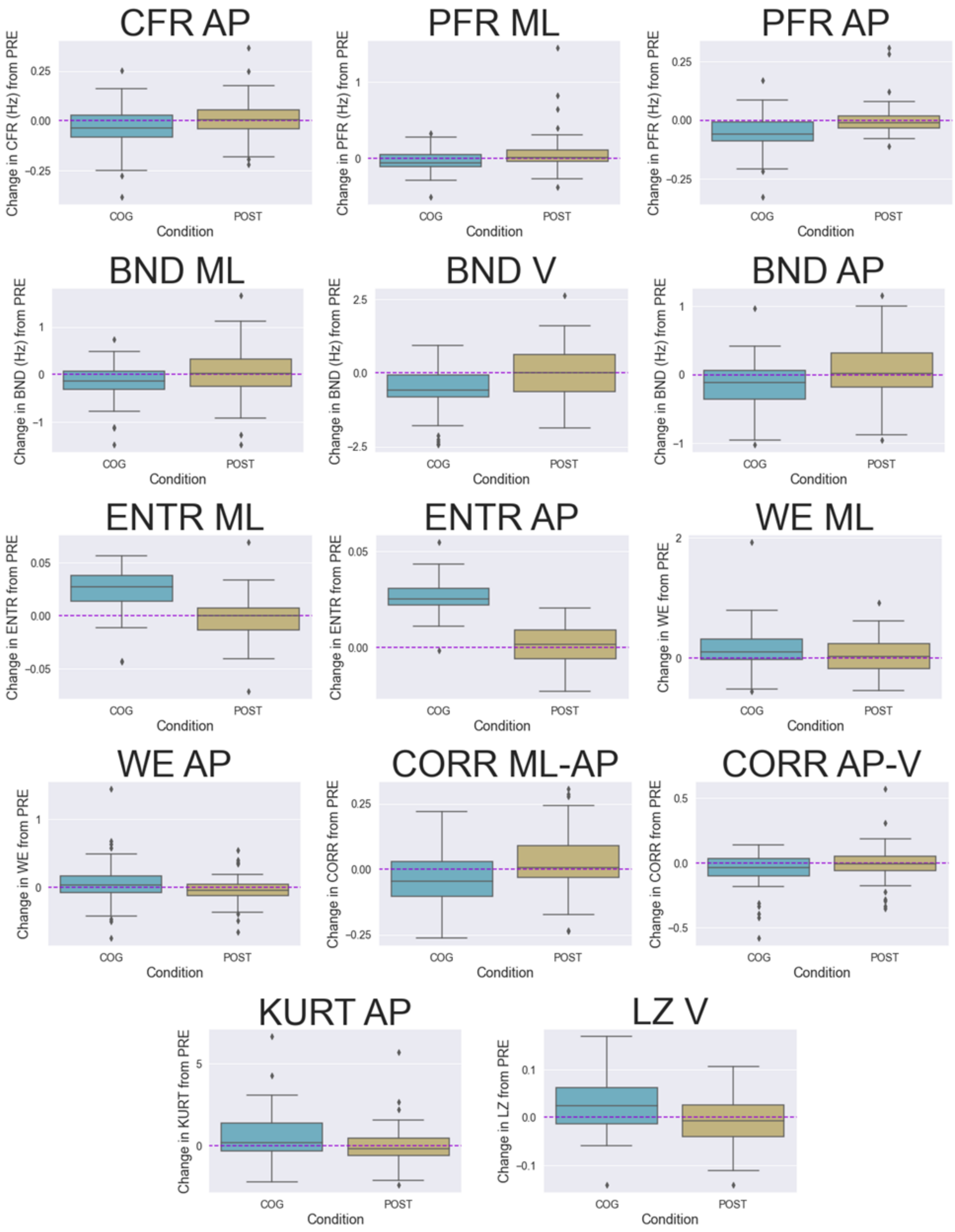

Figure S1. Box plots showing change from baseline for all significant variables. For each subject, average PRE values are subtracted from their averaged COG and POST values. Baseline, or initial values measured during the PRE condition, is indicated by the dashed line at 0. All variables except PFR ML and WE AP deviate during the COG condition and then return to baseline during the POST condition according to the results of the generalized linear regression model.
